# Quantification of Boat Visitation Rates at Artificial and Natural Reefs in the Eastern Gulf of Mexico Using Acoustic Recorders

**DOI:** 10.1371/journal.pone.0160695

**Published:** 2016-08-08

**Authors:** Peter Simard, Kara R. Wall, David A. Mann, Carrie C. Wall, Christopher D. Stallings

**Affiliations:** 1 University of South Florida, College of Marine Science, St. Petersburg, Florida, United States of America; 2 Loggerhead Instruments, Sarasota, Florida, United States of America; 3 University of Colorado, Cooperative Institute for Research in Environmental Sciences, Boulder, Colorado, United States of America; North Carolina State University, UNITED STATES

## Abstract

Artificial reefs are commonly used as a management tool, in part to provide ecosystem services, including opportunities for recreational fishing and diving. Quantifying the use of artificial reefs by recreational boaters is essential for determining their value as ecosystem services. In this study, four artificial–natural reef pairs in the eastern Gulf of Mexico (off western Florida) were investigated for boat visitation rates using autonomous acoustic recorders. Digital SpectroGram (DSG) recorders were used to collect sound files from April 2013 to March 2015. An automatic detection algorithm was used to identify boat noise in individual files using the harmonic peaks generated by boat engines, and by comparing the sound amplitude of each file with surrounding files. In all four pairs, visitation rates were significantly higher at the artificial reef than the natural reef. This increase in boat visitation was likely due to actual or perceived increased quality of fishing and diving at the artificial reefs, or to lack of knowledge of the presence or locations of the natural reefs. Inshore reefs (<15 m depth) had high variability in monthly visitation rates, which were generally highest in warmer months. However the seasonal signal was dampened on offshore reefs (>25 m depth). This study appears to be the first to use acoustic data to measure participant use of boating destinations, and highlights the utility of acoustic monitoring for the valuation of this important ecosystem service provided by artificial reefs.

## Introduction

Artificial reefs can provide hard-structured habitat that is used by reef fishes and other fauna, and consequently can provide ecosystem services for recreational, economic, and societal benefits to various stakeholder groups. The valuation of ecosystem services, such as those provided by artificial reefs, has become a paramount goal of ecosystem-based management of marine resources. The first necessary step in the valuation process of any resource is to quantify its use by involved stakeholders. For artificial reefs, this has been accomplished in several ways. For example, the analysis of dive shop log books and on-the-water observations indicated that the presence of two large artificial reefs in the Florida Keys (USS *Spiegel Grove* and USS *Vandenberg*) resulted in increased local participant use and associated economic benefits to area businesses [1,2]. In six southwestern Florida counties, boater surveys were used to estimate an annual use of artificial reefs of over 600,000 boat days [3].

While these studies have provided valuable information on the usage of artificial reefs, the methodologies have limitations. For example, on-the-water observations can only be conducted when surveys are logistically feasible for the research staff. Autonomous acoustic recorders may provide an alternative methodology for quantifying participant use of artificial reefs. The use of autonomous acoustic recorders as a remote sensing approach to measuring boat traffic has several advantages. Sound propagates farther in water than in air, therefore sounds tend to travel for long distances [4]. The noise created by recreational motorized vessels is high amplitude (e.g., typical peak narrowband source levels 150–165 dB re 1 μPa [5]) and typically low frequency (e.g., peak frequency at high RPM approximately 300–450 Hz [5]), therefore making signal loss from sound attenuation very low [4]. Therefore, passive acoustic monitoring of vessels is particularly effective and can operate at spatial scales of several kilometers. In addition, the use of autonomous recorders means that data collection can take place for long time periods, and by using multiple recorders, data can be collected simultaneously at multiple locations (thereby reducing temporal and spatial aliasing). The use of acoustics to identify boat noise has been a tool for military applications for decades, and its utility has been demonstrated in several studies and applications [6–12]. However, passive acoustic monitoring has not previously been used to compare the visitation rates at boating destinations for the purposes of value assessment of ecosystem services.

In this study, we quantified boat visitation rates at four artificial reefs paired with four neighboring natural reef sites in the eastern Gulf of Mexico using autonomous passive acoustic recorders. By measuring boat noise detections at different types of reefs at different locations, we could directly quantify resource use, which provides important information for the valuation of artificial reef ecosystem services related to recreational opportunities and economic benefits.

## Methods

**Fig 1:**
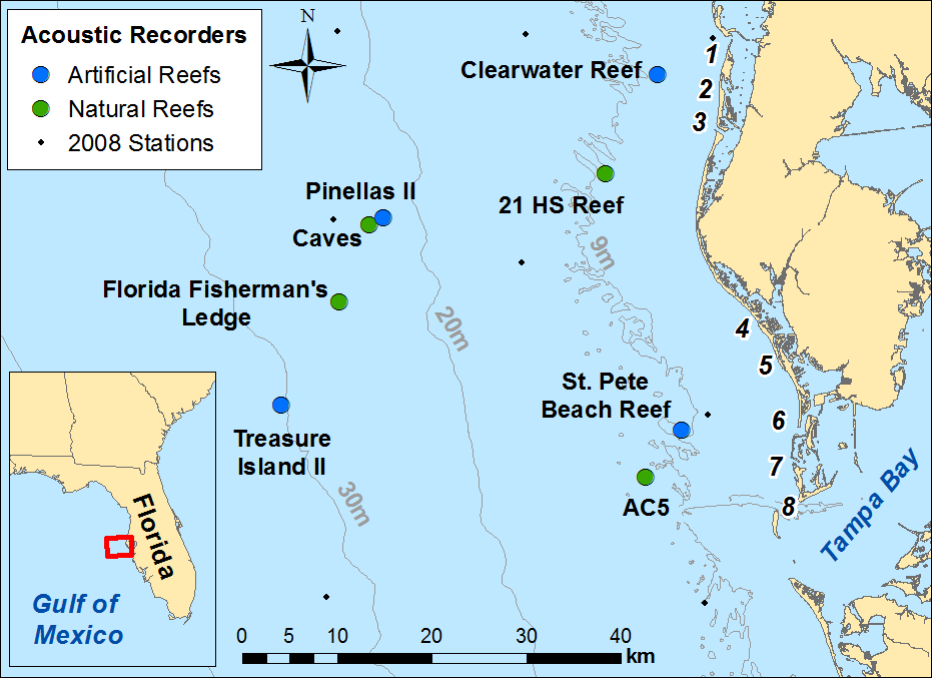
Map of study area. Map of study area, showing artificial and natural reef sites used in the study, and the locations of the 2008 recorders (recordings used in algorithm testing). Locations of passes shown by numbers: 1 = Hurricane Pass, 2 = Dunedin Pass, 3 = Clearwater Pass, 4 = John’s Pass, 5 = Blind Pass, 6 = Pass-a-Grille Pass, 7 = Bunces Pass, 8 = Egmont Channel. Data source for land and bathymetry: Florida Fish and Wildlife Conservation Commission—Fish and Wildlife Research Institute.

The study was conducted at eight reef sites in the eastern Gulf of Mexico, near Tampa Bay, Florida (Table 1, Fig 1). Four sites were artificial reefs established and maintained by the Florida Fish and Wildlife Commission Artificial Reef Program, and four were nearby natural reefs (limestone ledges). Therefore, each artificial reef investigated had a corresponding natural reef in a similar physical environment (e.g., currents, depth) and similar distance from passes used by boaters, to allow for a paired study design. No permissions were required to use these sites as the reefs are public resources, and field studies did not involve endangered or protected species. Although the reefs are monitored by the Florida Fish and Wildlife Commission Artificial Reef Program, monitoring cruises were infrequent (e.g., up to twice per month [13]) and therefore not expected to influence our results.

**Table 1:** Reefs investigated in study.

Pair	Reef	Reef Type	Latitude N	Longitude W	Depth (m)
1	Clearwater Reef	Artificial (concrete culverts, rubble)	28° 00.908’	82° 53.464’	9.8
	21 HS Ledge	Natural (limestone ledges, overhangs)	27° 55.372’	82° 56.847’	10.4
2	Pinellas II	Artificial (USCGS *Blackthorn*, other ships and boats, concrete rubble)	27° 52.753’	83° 10.994’	22.9
	Caves	Natural (limestone ledges, overhangs)	27° 52.167’	83° 12.023’	23.5
3	St Pete Beach Reef	Artificial (U.S. army tanks, concrete rubble)	27° 40.618’	82° 51.695’	10.0
	AC5	Natural (limestone ledges, overhangs)	27° 37.986’	82° 54.102’	12.8
4	Treasure Island II	Artificial (several ships and boats)	27° 41.681’	83° 17.315’	30.5
	Florida Fishermen’s Ledge	Natural (limestone ledges, overhangs)	27° 47.725’	83° 13.837’	25.9

Reefs investigated in study, reef type (artificial or natural, with a brief description of the predominant habitat types), position, depth.

Acoustic data were collected with DSG (Digital SpectroGram, Loggerhead Instruments) autonomous recorders. DSG recorders are self-contained, low power consumption, high sample rate acoustic recorders that have been used to monitor fish, marine mammals, and boat noise [12, 14, 15]. Data were recorded at 50 kHz sample rate and 16-bit resolution onto 32 GB SD cards. Hydrophones were HTI-96-MIN (High-Tech Inc., sensitivity: -170 dBV/μPa). Recorders operated on a duty cycle of 10 seconds every 10 minutes, allowing for a six month maximum service life (based on data storage on the SD card). Recorders were replaced every three months if logistics permitted (e.g., weather).

Each recorder was bottom mounted using concrete blocks (one central block with an imbedded galvanized eye for recorder attachment, and three additional blocks attached to the central block with 3 m lengths of duct-tape wrapped galvanized chain). The recorders were attached to 2 m ropes with stainless steel hose clamps. Each rope had a galvanized thimble spliced onto one end, which was situated to be at the bottom end of the recorder. The thimble-end of the rope was attached with a shackle to the galvanized eye in the central concrete block, allowing the recorders to float in a vertical (hydrophone up) orientation. In all cases, the DSG recorders were deployed approximately 200 m to the east (landward side) of the reef structure. This was done to reduce the risk of the recorders being tampered with by divers or being caught by boat anchors. The offset location of the recorders was not expected to decrease the number of boats detected, as the majority of boats visiting the sites would be transiting from the east (therefore passing over the recorders), and the offset distance was negligible in relation to typical boat sound propagation distances [4].

**Fig 2:**
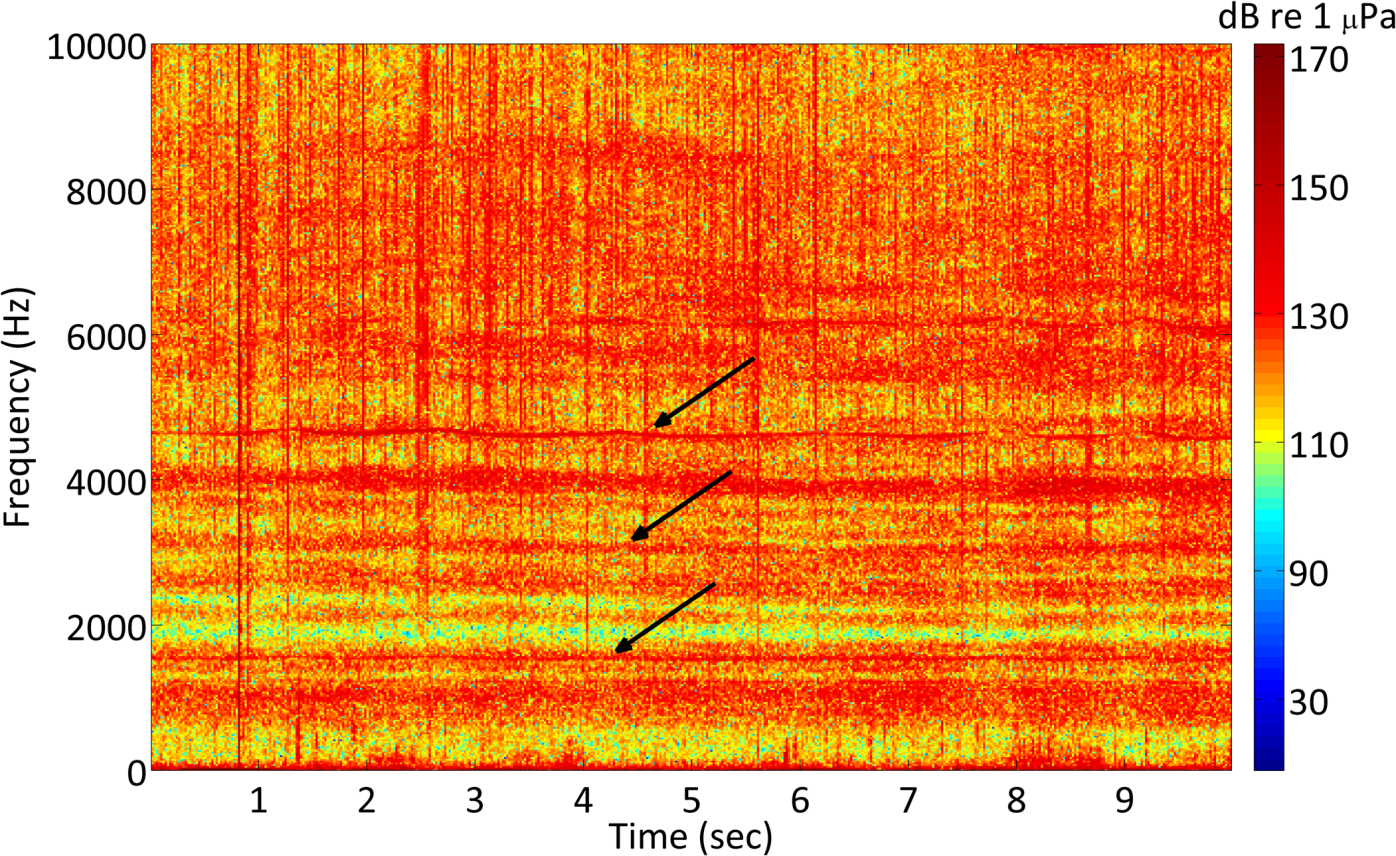
Spectrogram of boat noise. 2048 point resolution spectrogram of typical boat noise produced by an outboard engine driven boat at high speed. Arrows show several harmonics, which here extend upward to about 9000 Hz.

The large data set in this study made the manual inspection of spectrograms impractical, therefore the use of a boat detection algorithm was necessary. Boat noise is characterized by tonal harmonics caused by the cyclic properties of engine, shaft, and propeller rotation [9]. These harmonics appear as near-horizontal bands on a spectrogram (Fig 2) but are also digitally detectable by computers. A boat detection algorithm was developed and tested in MATLAB (version 2009b, Mathworks). The algorithm operated using five steps: (1) median filter (for background noise reduction, especially from impulse sounds from snapping shrimp and dolphin echolocation [16,17]), (2) band-pass filter (to reduce low frequency noise from fish chorusing [18], and to further reduce high frequency noise from snapping shrimp and dolphin echolocation), (3) FFT average (fast Fourier transform, to produce an averaged power spectrum of file), (4) peak identification (to identify harmonics typical of boat noise within averaged power spectrum), and (5) amplitude threshold (to determine if the overall root-mean-square [RMS, dB re 1 μPa, 500–1000 Hz] amplitude of the 10-second acoustic file was a threshold level above that of surrounding files).

The first four steps of the boat detection algorithm were tested and optimized in an iterative process using a test data set of 2,742 non-sequential acoustic files collected in 2008 in the same area as the study (Fig 1; 184 files with boat noise, 2,558 without boat noise). The amplitude threshold step was tested separately to improve algorithm performance using 2,282 sequential files containing 127 files with boat noise (sequential files were necessary in order to compare the amplitude of boat noise files with files recorded prior to and after vessel presence). All boat noise files were believed to be from recreational boats traveling at high speeds based on the presence of higher frequency harmonics [5], not large commercial vessels which have lower fundamental frequencies [11,19,20], although no visual confirmations were possible between acoustic recordings and boat sounds. For each algorithm trial, the proportion of all files correctly classified was determined (“correct classification rate”; files classified as boat noise when boat noise was present and files not classified as boat noise when no boat noise was present). Iterations were performed until the correct classification rate stabilized at a maximized value. Due to the fact that many more files were expected to not contain boat noise than contain boat noise, a conservative algorithm with a high correct rejection rate at the expense of a low detection rate was preferable (additional details on the boat detection algorithm testing are found in [21]).

To estimate the number of boats actually visiting each reef in a given time from the number of acoustic detections, the following formula was used:

$$\#\;boats=\left(\frac{D}{PDC*PAD}\right)*PV*(1-PFD)*SC*DC*0.5$$ (1)

Where D = mean detections per unit time (month) from the boat detection algorithm, PDC = probability of boat detection using the duty cycle, PAD = probability of correct boat detection by the automatic detection algorithm, PV = probability of a boat near a reef actually visiting the reef, PFD = probability of false boat detection by the automatic detection algorithm, SC = seasonal correction to account for different sound propagation in different water temperatures, and DC = depth correction to account for different sound propagation in different water depths. The last multiplier in the algorithm accounts for the fact that a boat is equally detectable as it leaves a reef as when it approaches a reef. Therefore, the estimate for the number of boats present must be halved.

The probability of boat detection using the duty cycle (PDC) was a measure of how effective the 10 second per 10 minute duty cycle was at “capturing” boat noise from a boat in acoustic detection range. In order to determine this variable, several recorders were programed to begin recording continuously near the end of their scheduled deployments. Ten 5-day long continuous files were analyzed. Boat sounds were manually detected by inspection of spectrograms of the continuous files using Adobe Audition (Adobe Systems, Inc.), and a 10 second per 10 minute duty cycle was simulated for each.

The probability of a boat close to a reef that was actually visiting the reef (PV) was determined by field observations during recorder servicing cruises (approximately once every three months). At each reef site, the number of boats which stopped for any amount of time at the reef was recorded, as well as the number of boats which passed over or near the reef without stopping (within approximately 1 km) was recorded during each visit. From this data, the overall proportions of boats stopping at each reef were calculated (number of boats stopping / total number of boats).

As sound propagation changes with water temperature and thermal stratification, as well as with depth [4], the potential differences in detection probability with different seasons and depths needed to be addressed (seasonal correction: SC; depth correction: DC). To investigate this problem, boats traveling at high speeds were recorded (from the research vessel or personal vessels), along with information regarding the type and size of the vessel being recorded, and the estimated distance from the hydrophone (using a laser range finder when possible, or estimation of distance after an observer training session, see [21] for details). Boat sounds were recorded in all months of the year during the study period, and in depths ranging from 8 m to 30 m (i.e., the approximate depth range of our study sites). To investigate maximum potential received level differences, recordings made in the coldest water temperatures (December–February) were compared with those made in the warmest water temperatures (July–September). Recordings were further classified by depth range, shallow (8–12 m), and deep (>20 m). From field observations, the size and type of vessel with the maximum number of recordings in all seasons and depths was the center console, single engine, 25–29’ (7.6–8.8 m), and recordings were most commonly made at a distance of 200–300 m. Therefore, for this boat type and range, the received levels were compared for inshore and offshore in warm water temperatures, and inshore and offshore for cold water temperatures.

Only full recording days were analyzed in this study; partial days of recording due to DSG recorder deployment or recovery were omitted from analysis in order to eliminate detections of the research vessel. Using the boat noise detection algorithm and the detections-to-boats algorithm, the mean number of boats per day at each reef was calculated for each month of the study.

Boat detection rates were analyzed to determine if artificial reefs and natural reefs received different numbers of visiting boats during the study. It was expected that there would be considerable amounts of inter-month variability in boat visitations. Therefore, only comparisons between reefs using shared months (months recorded at both reefs) were used for statistical analysis. There was also expected that there would be more shared months of data between two recorders than between all eight; therefore, to maximize the number of shared months for analysis, individual two-tailed t-tests were performed on each artificial–natural reef pair. In order to determine if monthly boat visitation rates of each individual reef varied with month, we used Chi-squared goodness of fit tests to determine if the visitation rates were different than a null-hypothesis uniform distribution.

## Results

The optimized values for the boat-detection algorithm were a 10-point median filter, a 500–7,000 Hz band-pass filter, an FFT average of 300 Hz, and a threshold for peak identification of four peaks. The amplitude threshold optimum was found using a five-file window (two files before and two files after the file being analyzed) and 0.5 standard deviation above the mean RMS amplitude. Using these values, the algorithm had a correct detection rate of 11.8% and a correct rejection rate of 99.5%.

For the variables in the detections-to-boats algorithm, the mean probability of boat detection using the duty cycle (PDC) was 0.543 (SD = 0.064). There was no significant difference in PDC between artificial reefs and natural reefs (ANOVA, F = 0.582, p = 0.467) or inshore and offshore reefs (ANOVA, F = 0.033, p = 0.861). Therefore, the mean value of 0.543 was used as the probability of duty cycle detection for all stations. For the probability of boats vising the reef (PV), values appeared to be reef specific, with artificial reefs generally being higher. Therefore, the algorithm used reef specific values for PV (Clearwater Reef = 0.619, 21 HS Ledge = 0.111, Pinellas II = 0.714, Caves = 0.111, St. Pete Beach Reef = 0.750, AC5 = 0.111, Treasure Island II = 0.667, Florida Fisherman’s Ledge = 0.200). There were no significant differences between the received levels in different seasons or different depths (ANOVA, F = 0.216, p = 0.885). Therefore, neither depth nor season were considered to be significant factors in detection probabilities, and SC and DC were not included in the calculations. This conclusion was supported by previous work that found that sound propagation varied little in the Gulf of Mexico over depths similar to the current study [22].

**Fig 3:**
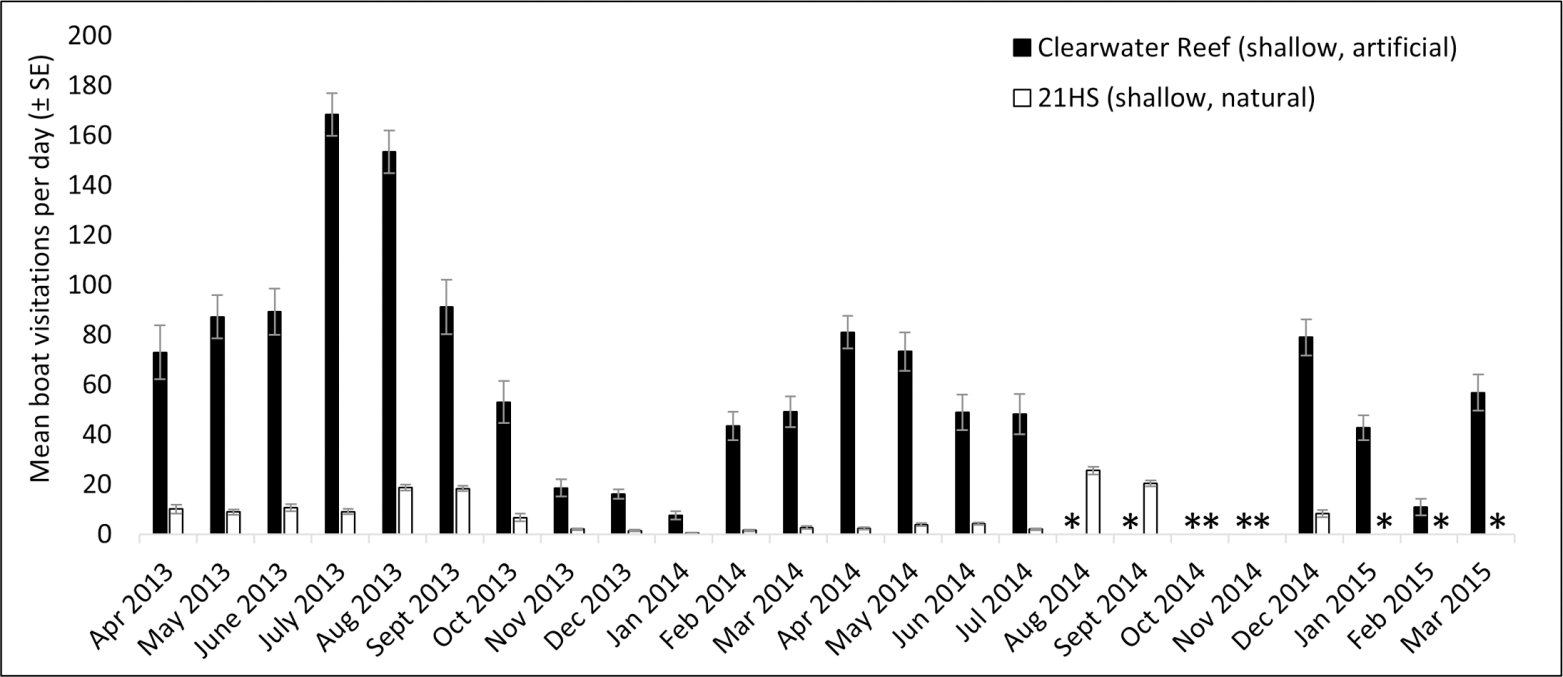
Mean visitation rates for Clearwater / 21 HS. Mean boat visitation rates (± SE) for Clearwater artificial reef and 21 HS Ledge natural reef. “*” indicates missing data.

**Fig 4:**
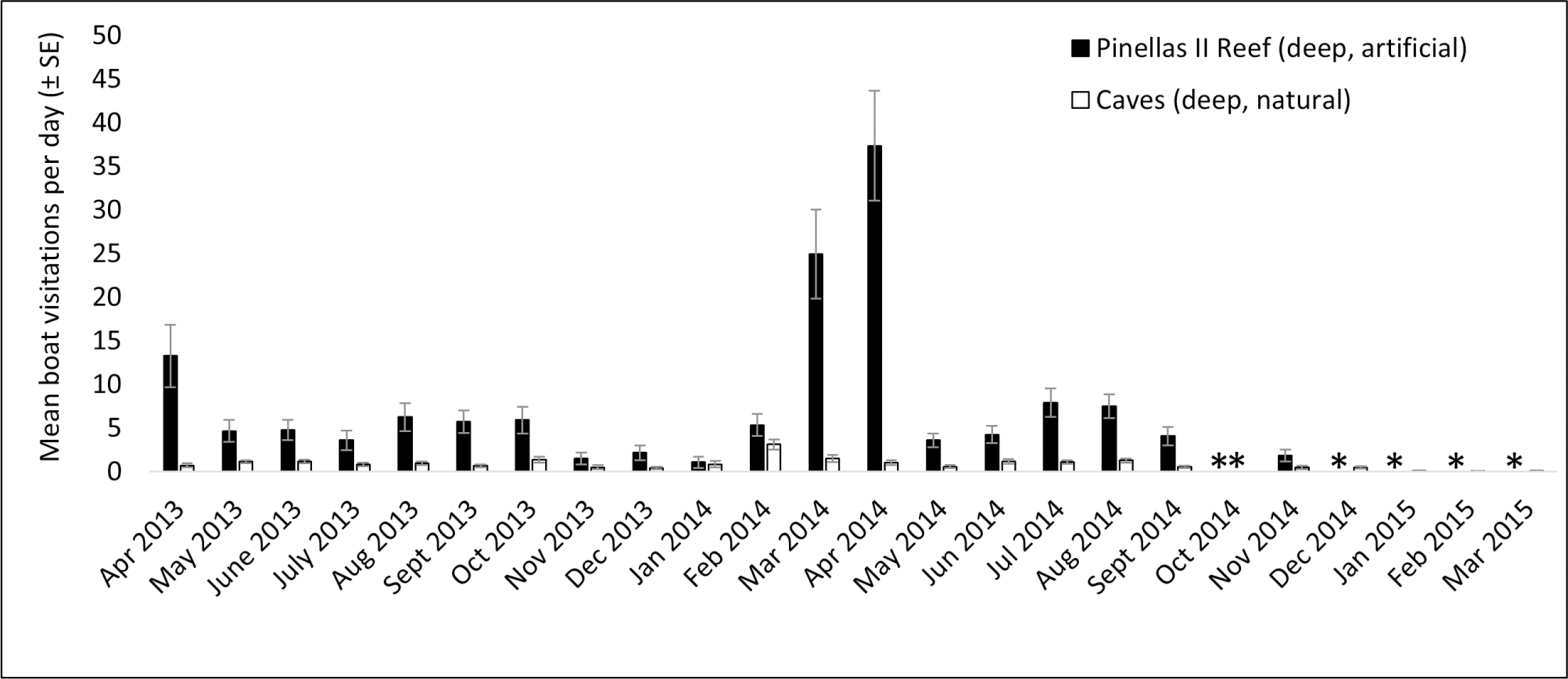
Mean visitation rates for Pinellas II / Caves. Mean boat visitation rates (± SE) for Pinellas II artificial reef and Caves natural reef. “*” indicates missing data.

**Fig 5:**
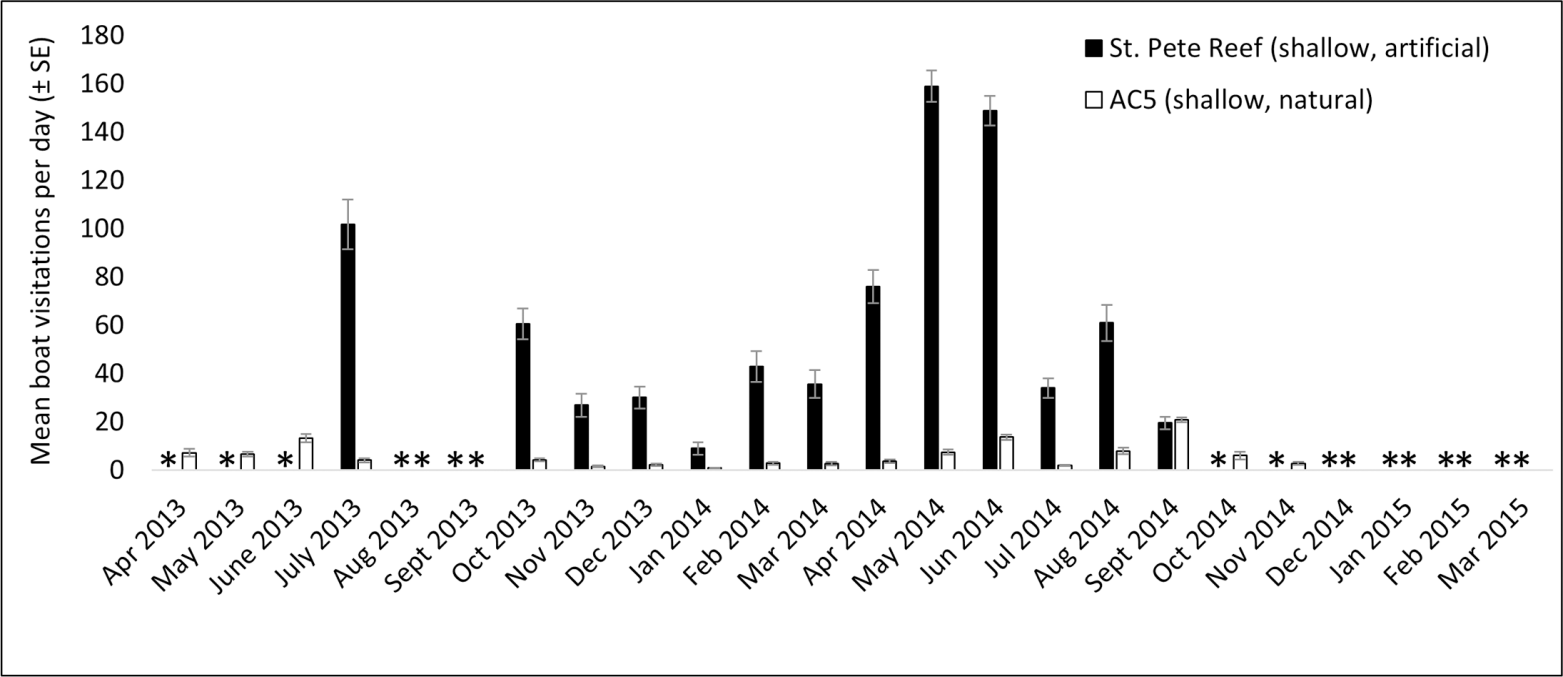
Mean visitation rates for St. Pete Reef and AC5. Mean boat visitation rates (± SE) for St. Pete artificial reef and AC5 natural reef. “*” indicates missing data.

**Fig 6:**
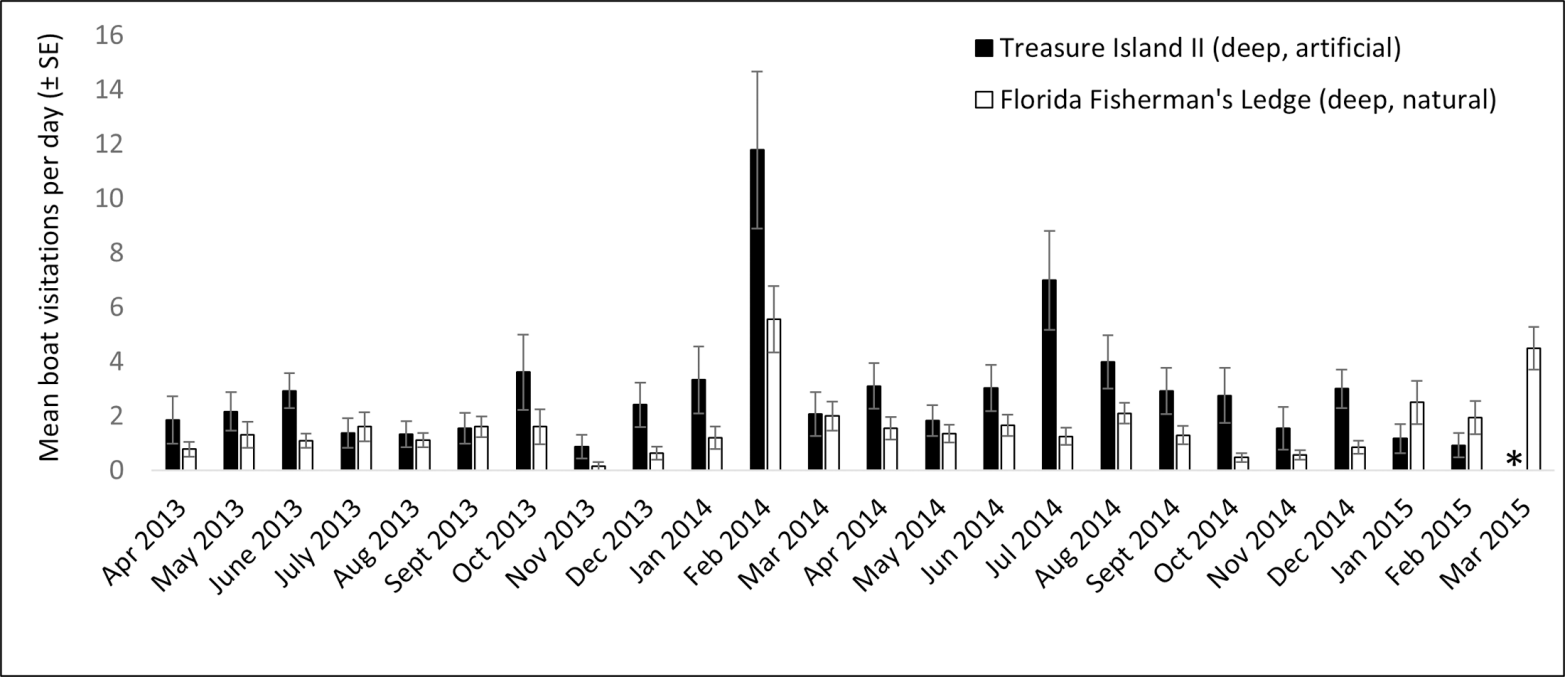
Mean visitation rates for Treasure Island II / Florida Fisherman’s Ledge. Mean boat visitation rates (± SE) for Treasure Island II artificial reef and Florida Fisherman’s Ledge natural reef. “*” indicates missing data.

Acoustic data were collected between April 2013 and March 2015, resulting in 4,585 days and 660,240 files for analysis (see S1 Appendix–S8 Appendix). Monthly averages for the daily boat visitation rates were calculated for each station (Figs 3–6). Acoustic data collection was interrupted in several cases due to recorder failure or delayed recorder switch-outs due to inclement weather and other logistical challenges in accessing the sites. In all artificial–natural reef pairs, the artificial reef had significantly higher boat visitation rates than the natural reef (Table 2).

**Table 2:** Boat visitation rates.

Reef Pair	Reef	Mean	SE	t	p	df
1	Clearwater	69.38	10.46	6.617	< 0.001	16
	21 HS Ledge	6.49	1.37			
2	Pinellas II	8.20	2.14	3.263	0.004	18
	Caves	1.05	0.14			
3	St. Pete Beach Reef	66.27	14.20	4.373	0.001	12
	AC5	5.63	1.59			
4	Treasure Island II	2.89	0.49	3.840	0.001	22
	Florida Fisherman’s Ledge	1.48	0.22			

Mean, standard error (SE) and t-test results for differences in boat visitation rates

Monthly boat visitation rates were significantly variable for all four inshore reef sites: Clearwater Reef (artificial reef, χ^2^ = 518.3, p < 0.001, df = 19), 21 HS Ledge (natural reef, χ^2^ = 116.8, p < 0.001, df = 18), St. Pete Beach Reef (artificial reef, χ^2^ = 438.0, p < 0.001, df = 12), and AC5 (natural reef, χ^2^ = 67.5, p < 0.001, df = 16). In all cases, there was a tendency for increased boat visitation rates in warmer months, and greater within-month variation was observed during warmer months. Less variation in monthly boat visitation rates was observed in the four offshore reef sites. Significant variation was observed at Pinellas II (artificial reef, χ^2^ = 141.1, p < 0.001, df = 18), but not at Caves (natural reef, χ^2^ = 9.6, p = 0.990, df = 22), Treasure Island II (artificial reef, χ^2^ = 28.0, p = 0.176, df = 22), or Florida Fisherman’s Ledge (natural reef, χ^2^ = 15.0, p = 0.927, df = 23). At these offshore reef sites, large increases in boat visitation rates were observed in spring (February—April).

## Discussion

Four artificial–natural reef pairs in the eastern Gulf of Mexico were investigated for boat visitation rates: Clearwater Reef (inshore, artificial) and 21 HS (inshore, natural), Pinellas II (offshore, artificial) and Caves (offshore, natural), St. Pete Beach Reef (inshore, artificial) and AC5 (inshore, natural), and Treasure Island II (offshore, artificial) and Florida Fisherman’s Ledge (offshore, natural). In every artificial–natural reef pair studied, significantly more boats visited the artificial reef site than the natural reef site. At the inshore locations, visitation rates at the artificial reef sites were approximately ten times higher than those at natural reefs. Differences in visitation rates were approximately eight times higher at the offshore artificial reef Pinellas II than the natural reef Caves; however, Treasure Island II artificial reef only had approximately twice the boat visitations than its paired natural reef, Florida Fisherman’s Ledge. The proportions of boats visiting the reefs (PV in Eq 1) also suggest higher visitation rates at artificial reefs, as the proportion of boats visiting artificial reefs was much higher than for natural reefs (artificial reefs 0.619–0.750, natural reefs 0.111–0.200). There appear to be few investigations on the amount of boat traffic on artificial reef sites in in comparison to natural reef sites. To our knowledge, the only previous comparisons were on the artificial reefs USS *Spiegel Grove* and USS *Vandenberg* [1,2]. In these studies, the establishment of the USS *Spiegel Grove* artificial reef reduced diver visitations at nearby natural reefs by 13.7% despite an overall increase in diving in the area, although this was not the case for the USS *Vandenberg* [1,2]. The popularity of artificial reefs has been observed in various locations (e.g., Texas [23]), and both the high levels of use and the economic importance of artificial reefs off western Florida have been well documented [3,24].

The high rates of boat visitation at artificial reefs in comparison to natural reefs in this study are likely due to increased recreational value perceived by sport fishers. Fishing was a very common activity among boaters at the artificial reefs in this study (personal observation). Most boaters (64%) in the Tampa Bay area (which would include boaters potentially visiting the reefs used in our study) fished during their boating trips, and the most common reason for selecting boating destinations was better fishing opportunities (35.6% of surveyed boaters [25]). The ecological role of artificial reefs on fish population dynamics is controversial [26, 27], and some important recreational species were found in lower density and/or biomass at our artificial reef sites than at the natural reef pair (e.g., red grouper, *Epinephelus morio* [21]). However, recreational fishermen often report high success rates on artificial reefs [23,27], and the opinion that artificial reefs increase the amount of desirable species is shared by most users in Florida [24].

Diving, and particularly spearfishing, may also be an important activity at the artificial reefs in the study region, and likely contributes to the increased boat visitation rates at these reefs. Dive flags and / or divers in the water were frequently observed at the artificial reef sites (personal observation). Artificial reefs have been found to be popular with divers in several studies [1,24,28,29], possibly due to actual or perceived spearfishing success, nature viewing, or other ecosystem services [29]. Artificial reefs composed of large ships and other large structures are known to attract divers [1,23,28], and likely attract traditional sport divers, not just those interested in spearfishing. Several large wrecks are found at the offshore artificial reefs in this study (e.g., the USCGC *Blackthorn* and the *Sheridan* tug at Pinellas II, and two commercial fishing boats at Treasure Island II), which are popular destinations for local diver charters (personal observation).

Another factor in the higher boat visitation rates at artificial reefs may be that the presence and locations of the reefs are simply better known than the natural reefs. Access to location information has been found to be an important factor in determining boater visitation patterns in several studies [23,29]. The locations of the artificial reef sites in this study are published in various locations. For example, the artificial reefs are highlighted in the Florida Fish and Wildlife Commission Artificial Reef Program website [30] and the popular fishing website www.floridagofishing.com [31]; however, these sites contain no information on the natural reefs used in this study. Additionally, dive stores local to the study region were knowledgeable about the artificial reefs in this study, but were only vaguely familiar with any natural reefs (personal observation).

All inshore reef sites had significant monthly variability, with visitation rates tending to peak in warmer months. This result generally reflects the temporal patterns observed in boating activity, which has previously been found to peak in April through August in the study region [25]. At the offshore reef sites, monthly variation was only significant at Pinellas II artificial reef. This may be due to the fact that Pinellas II had higher visitation rates than other offshore reefs, possibly making monthly variability more pronounced. A peak in boat visitations was observed in the offshore reefs in spring (February–April). These peaks could potentially be due to commercial fishing activity. Reef fish catch data from federal waters (> 9 nm, 16.7 km) off west-central Florida indicate higher levels of fishing from February through May 2014 [32]. Although it is not possible to determine the locations of this fishing activity, it is possible that the peaks in boat detections within this time period at the deep water reefs are at least partially explained by commercial fishing activity. These peaks, if caused by commercial vessel activity, could potentially be eclipsing the underlying seasonal patterns in recreational boating activity.

Several improvements could be made to the methodology of this study for future efforts to quantify boat visitation rates using passive acoustics. The detection algorithm used to identify boat noise in acoustic files could potentially be improved with additional testing (with larger data sets for example) or a learning algorithm. The background noise levels in the study area were highly variable. In a previous study, ambient noise in the study area was found to be mainly due to boat noise and snapping shrimp, both of which increased during warmer months and in shallower waters [15]. In this study, as the boat noise itself was the desired signal, spatial and temporal trends in snapping shrimp noise could influence boat detection rates, and further investigation of this potential bias is warranted, especially as the periodicity of snapping shrimp acoustic activity appears to be temporally complex [33]. High levels of ambient noise created many challenges for the design of a successful boat noise detector as signal detection in high-noise environments is generally a more difficult task than in quiet environments. Largely because of this, in order to not have the signal (correct boat detections) eclipsed by the noise (false boat detections), the parameters of the algorithm were adjusted so that the algorithm performed very conservatively. Therefore, in high-noise environments, improved noise-reduction steps would be advantageous. Although we found no significant difference in boat noise between depths and seasons, differences may exist and a more thorough investigation would be worthwhile.

This study was one of the first to directly compare the use of artificial reefs to the use of nearby natural reefs, and the first to do so using passive acoustics. Using passive acoustic monitoring to measure boat traffic is a practical method in that it allows for synoptic-scale data collection. Multiple sites can be simultaneously monitored, “continuously” (with a given duty cycle in this case) for long time periods. This allowed for the assessment of boat visitation rates over large spatial and temporal scales. The results of this study indicate that boat visitation rates at four artificial reefs in the eastern Gulf of Mexico are significantly higher than boat visitation rates at nearby natural reef sites. Our results support a growing body of evidence indicating that artificial reefs provide considerable cultural ecosystem services [3]. In addition, given the inherent advantages of the methodology, passive acoustic monitoring should be seriously considered by coastal managers in order to quantify such ecosystem services.

## Supporting Information

S1 Appendix Boat detection algorithm output for Clearwater artificial reef. Appendix includes the station name, the folder name in the original analysis, the file number of the recording, the time (Eastern Standard Time) and date of the recording, the number of harmonic peaks detected by the algorithm, and the root-mean-square noise level (RMS, dB re 1 μPa, 500–1000 Hz). Each tab contains data from three months of the study.
(XLSX)

S2 Appendix Boat detection algorithm output for 21 HS natural reef. Appendix includes the station name, the folder name in the original analysis, the file number of the recording, the time (Eastern Standard Time) and date of the recording, the number of harmonic peaks detected by the algorithm, and the root-mean-square noise level (RMS, dB re 1 μPa, 500–1000 Hz). Each tab contains data from three months of the study.
(XLSX)

S3 Appendix Boat detection algorithm output for Pinellas II artificial reef. Appendix includes the station name, the folder name in the original analysis, the file number of the recording, the time (Eastern Standard Time) and date of the recording, the number of harmonic peaks detected by the algorithm, and the root-mean-square noise level (RMS, dB re 1 μPa, 500–1000 Hz). Each tab contains data from three months of the study.
(XLSX)

S4 Appendix Boat detection algorithm output for Caves natural reef. Appendix includes the station name, the folder name in the original analysis, the file number of the recording, the time (Eastern Standard Time) and date of the recording, the number of harmonic peaks detected by the algorithm, and the root-mean-square noise level (RMS, dB re 1 μPa, 500–1000 Hz). Each tab contains data from three months of the study.
(XLSX)

S5 Appendix Boat detection algorithm output for St. Pete Reef artificial reef. Appendix includes the station name, the folder name in the original analysis, the file number of the recording, the time (Eastern Standard Time) and date of the recording, the number of harmonic peaks detected by the algorithm, and the root-mean-square noise level (RMS, dB re 1 μPa, 500–1000 Hz). Each tab contains data from three months of the study.
(XLSX)

S6 Appendix Boat detection algorithm output for AC5 natural reef. Appendix includes the station name, the folder name in the original analysis, the file number of the recording, the time (Eastern Standard Time) and date of the recording, the number of harmonic peaks detected by the algorithm, and the root-mean-square noise level (RMS, dB re 1 μPa, 500–1000 Hz). Each tab contains data from three months of the study.
(XLSX)

S7 Appendix Boat detection algorithm output for Treasure Island II artificial reef. Appendix includes the station name, the folder name in the original analysis, the file number of the recording, the time (Eastern Standard Time) and date of the recording, the number of harmonic peaks detected by the algorithm, and the root-mean-square noise level (RMS, dB re 1 μPa, 500–1000 Hz). Each tab contains data from three months of the study.
(XLSX)

S8 Appendix Boat detection algorithm output for Florida Fisherman’s Ledge natural reef. Appendix includes the station name, the folder name in the original analysis, the file number of the recording, the time (Eastern Standard Time) and date of the recording, the number of harmonic peaks detected by the algorithm, and the root-mean-square noise level (RMS, dB re 1 μPa, 500–1000 Hz). Each tab contains data from three months of the study.
(XLSX)
